# Opinion: can we bust the fear of symptomatic intracerebral hemorrhage due to tPA?

**DOI:** 10.3389/fneur.2024.1428726

**Published:** 2024-09-19

**Authors:** Adalia H. Jun-O'Connell

**Affiliations:** Department of Neurology, UMass Chan Medical School, Worcester, MA, United States

**Keywords:** IV thrombolysis, alteplase, tenecteplase, symptomatic intracerebral hemorrhage (sICH), thrombolysis complications

## Introduction

Since the publication of the landmark NINDS trial (11) of tissue plasminogen activator (tPA) effect on acute ischemic stroke, IV thrombolysis in select acute ischemic strokes has become a standard of care, and it is considered a crucial treatment that can improve the long-term functional outcome in stroke (1). Its efficacy in ischemic stroke is well known, and yet its usage is known to be limited by its risk of hemorrhagic complication (2). The fear of potential tPA related symptomatic intracerebral hemorrhage (sICH) still persists in the community (3), and tPA remains underutilized in eligible populations (4) even though the literature supports that the benefits of the thrombolysis outweighs the hemorrhagic risk and improves long-term outcomes (5–14). IV thrombolysis utilization among adults with acute ischemic strokes increased over time between 2002 and 2015, and yet only 1 in 15 acute ischemic strokes were found to receive IV thrombolysis; inequities were most noticeable for African-Americans, women, those treated in rural areas, and the government insured (4). In the USA, physician discomfort or hesitancy surrounding tPA is one of the major issues attributing to limited IV thrombolysis utilization (15). Furthermore, universally low IV thrombolysis rates (overall 7.3%) are also observed in European countries for similar reasons as in the USA (16). These reasons include numerous relative contraindications; narrow treatment time windows; provider uncertainty about patient eligibility; the heterogeneity in the efficacy of local health systems; expertise stroke care resource availability; and variations in stroke awareness and geographical barriers (16). Subsequently, a review of the landmark randomized controlled tPA trials in ischemic stroke and large patient stroke registry data is necessary, with the aim to understand that tPA usage in select ischemic strokes is indeed safe. A review on thrombolysis related sICH is relevant since it provides a safety review regarding tPA in acute ischemic stroke (17). However, there are three important points to be recognized regarding the review of the RCTs on IV thrombolysis. One, the perception that sICH due to tPA is increased by “6-fold [depending on the definition used]” (17) needs to be carefully interpreted as the benefit of the treatment far outweighs the potential complication (18–20). Two, the incidence of sICH due to IV thrombolysis can vary across stroke studies, thus consistent definition needs to be utilized through the Stroke Centers. And three, a larger pool of population data needs to be further publicized to reflect the real-life occurrence of tPA relevant sICH.

## Discussion

Net functional benefit of thrombolytic therapy is well-known in ischemic stroke, especially in improvement of long-term functional outcome, as demonstrated by multiple trial results (19, 21–26). IV thrombolysis remains the first-line treatment option for eligible patients in an acute ischemic stroke, which is one of the major causes of worldwide mortality and morbidity (27). It is also associated with the potential risk of symptomatic intracerebral hemorrhage complication, with the most symptomatic hemorrhages known to occur within the first 12 h of the treatment (28). Despite its known risk, it is the only approved systemic reperfusion therapy, proven effective in improving outcomes and reversing stroke deficits (29). It must be emphasized that even though there is a concern for a potential harm, the treatment's benefit outweighs the feared complication from tPA. Specifically, it was noted that for every 100 acute ischemic stroke patients treated with tPA, 32 would benefit and 3 would be harmed as a result of tPA-related sICH (18, 30). ECASS 3 trial also noted that the net benefit of the IV thrombolysis was higher than the net harm, with the likelihood of help to harm ratio being 6.0 (7, 20). Therefore, IV thrombolysis in ischemic stroke is justified and needs to be supported given the magnitude of the benefit attributed to the treatment.

### sICH due to tPA is low

First, it is important to remember that sICH due to tPA is considered low. Further review of the article referenced by Maier, Desilles, and Mazighi shows a meta-analysis of RCTs comparing alteplase vs. placebo in acute ischemic stroke that found that the sICH due to tPA is 3.7% vs. 0.6% (comparing 3,391 vs. 3,365 pts; OR 6.67), and that the fatal ICH is 2.7% vs. 0.4% (OR 7.14) (31). Although a 6-fold increase in risk may be considered drastic, the risk percentage despite the increase is small.

Based upon the meta-analysis of IV thrombolysis studies from 1994 to 2011, the risk of sICH varied based on the patient population and the definition of sICH used (2), but is known to range between 2% to 7% (2). Significant differences were observed depending on the study design, with higher sICH rates observed in randomized control trials (mean 7.45%) compared to lower rates in stroke registries (mean 3.5%) (32). Three important observations were highlighted that explain the difference: (1) the incidence of sICH due to IV thrombolysis varied across stroke studies and according to differences in the sICH definition criteria; (2) these differences resulted in inconsistencies in sICH rate; and (3) the highest consistency of sICH rate was observed in the cohort studies and in the studies that defined sICH as “parenchymal hemorrhage associated with NIHSS increments of ≥4 points occurring within 36 h of IV thrombolysis” (32).

### Definition of sICH due to tPA has evolved over time

Second, since the inception of NINDS (11) and ECASS (12) RCTs, the definition of tPA relevant ICH has changed over time. Currently, Stroke Centers following Joint Commission standards use the most up-to-date definition of tPA relevant sICH as a “symptomatic intracerebral hemorrhage (i.e., clinical deterioration ≥4 point increase on NIHSS and brain image finding of parenchymal hematoma, or subarachnoid hemorrhage, or intraventricular hemorrhage) within ( ≤ ) 36 h after the onset of treatment with intra-venous (IV) or intra-arterial (IA) alteplase therapy, or mechanical endovascular reperfusion procedure (i.e., mechanical endovascular thrombectomy with a clot retrieval device)” (33).

Various definitions of sICH have been used in IV thrombolysis trials over the years (34, 35). From a radiological perspective, the definition of asymptomatic vs. symptomatic ICH may appear arbitrary. ECASS I and II are examples of the radiological definition of hemorrhagic transformation in which the parenchymal hematoma (PH) greater than 30% of the infarcted area with a significant space occupying mass effect was associated with a poor neurological outcome (35, 36). The effect of clinically asymptomatic hemorrhagic transformation (HT) on stroke outcome also remains controversial, but overall, it is considered to not have a negative effect in IV thrombolysis (35–37). The *post-hoc* analysis of the ECASS I also noted that hemorrhagic infarcts or PH with only mild space occupying effect (HI 1, 2, and PH-1) or petechial hemorrhage did not worsen post stroke mortality or clinical deterioration (36). Furthermore, an acute neurological deterioration following the IV thrombolysis may also occur due to reasons other than the hemorrhagic transformation, such as the size of the stroke (35).

It is well known that hemorrhagic transformation of ischemic stroke is a common phenomenon; a meta-analysis of 17,259 ischemic patients demonstrated, for example, an overall prevalence of 27% (35). Based on that same meta-analysis, it is also known that hemorrhagic transformation is higher amongst patients that received IV thrombolysis vs. without (32% vs. 20%) (35). It is also known that hemorrhagic transformation occurs up to 42% in acute ischemic strokes, and more than half of all cerebral infarcts cause certain stages of hemorrhagic transformation (38). Considering the dilemma of various definitions used in the trials for IV thrombolysis and hemorrhagic complication, a pragmatic approach needs to be considered for stroke systems of care. The SITS-MOST definition of sICH offers the most thoughtful approach, as its definition considers an acute neurological deterioration plus radiological change (HT) within the specific time period from the IV thrombolysis (39). The SITS-MOST definition is also considered stricter, as it includes radiological change of PH2 formation, plus the specific clinical deterioration definition during the time frame of 22–36 h, especially as IV thrombolysis hemorrhages are known to occur within the first 24 h (34).

When comparing definitions, one could argue that the later studies, including ECASS 3 (7) and SITS-ISTR (8), share a similar refined definition of sICH (7, 8). Overall, it is known that the ECASS II definition has the highest interrater agreement, whereas the SITS-MOST definition correlates most strongly with poor outcomes and mortality (2, 34). In the standard clinical settings in which American Stroke Centers are expected to follow the Joint Commission standards of stroke care, it is recommended that when reporting and interpreting sICH due to IV thrombolysis, the HT is classified according to the radiological criteria and the degree of neurological worsening is assessed by NIHSS, and that the stroke centers provide an attribution of causality for the worsening neurological status (2). Based upon the later definitions, symptomatic ICH due to tPA was indeed found to be low. For example, the results of ECASS-III trial (7) indicated that sICH due to tPA is 2.4% (7). According to the updated analysis from SITS-ISTR (43), which was a large observational study on tPA (*N* = 23,942), sICH due to tPA was 1.7% if given within 3 hours from onset vs. 2.2% if given between 3- to 4.5-hr window using SITS-MOST definition of sICH (43).

Even during the time of transition from alteplase to tenecteplase, it is important to note that the risk of sICH due to tPA was still found to be low. The EXTEND-IA TNK (14) trial, which compared alteplase to tenecteplase, found 1% sICH in each group (*N* = 202). The ACT (42) trial, which was even larger (*N* = 1577), showed 3.4% vs. 3.2% sICH (TNK vs. alteplase).

In RCTs that also offered tPA outside the four-and-a-half-h window, the symptomatic ICH due to tPA was reassuring: 2% tPA vs. 0.4% placebo in the WAKE-UP trial [*N* = 503, (6)]; and 6.2% tPA vs. 0.9% placebo in the EXTEND trial ((5), *N* = 225) (5). Higher tPA complication is expected in an extended tPA window (21).

### Larger population registry data results need to be further shared

The American Heart Association's “Get with the Guidelines” (GWTG) registry data published in 2015 showed 58,265 ischemic strokes that received tPA from 2009 to 2013. The publication found the overall incidence rate of sICH due to tPA was 4.7% (40). The incidence rate is further decreasing over the recent years. In a recent cohort study of 321,819 ischemic stroke patients from GWTG-Stroke registry who received IV thrombolysis from 2013 to 2021, the overall rate of sICH was 3.3% (41).

## Conclusion

In conclusion, the fear of sICH due to tPA needs to be tempered with the fact that overall tPA relevant sICH is low, and that the benefit of the tPA in improving post stroke functional outcome is known to be significant. Furthermore, the definition of sICH has evolved over time, and the population registry data has been reassuring.
